# Association of Practices Regarding Infant and Young Child Feeding with Anthropometry Measurements Among an Urban Population in Karnataka, India

**DOI:** 10.7759/cureus.4346

**Published:** 2019-03-29

**Authors:** Bhanuja Bhagwat, Shalini Chandrashekar Nooyi, Dinesh H Krishnareddy, Srinivasa Nandagudi Murthy

**Affiliations:** 1 Department of Community Medicine, M S Ramaiah Medical College, Bangalore, IND

**Keywords:** initiation of breastfeeding, continued breastfeeding, anthropometry, community, complementary feeding, gripe water, iycf, urban slum, mgrs, nfhs

## Abstract

Background

Mothers' knowledge of infant and young child feeding (IYCF) play a crucial role in the overall growth and development of a child, determined by their anthropometry. Malnutrition has been linked to the short- and long-term effects on child health and, ultimately, national development. This community-based cross-sectional study focuses on the nutritional parameters of infants and young children in an urban slum population. The primary objective was to study IYCF from birth up to two years of age. The secondary objective involved studying the association between feeding practices and anthropometric measurements among children of 12-23 months of age.

Methods

The mothers of 96 children who were between 12 and 23 months of age, living in the urban slum of B.K Nagar, Bangalore, India, were administered the United Nations Children's Fund (UNICEF) IYCF questionnaire. Breastfeeding and complementary feeding information from these children from birth to 24 months were obtained. Anthropometric measurements were measured with appropriate calibrations.

Results

The population consisted of 57.3% females and 42.7% males. All children had mean anthropometric measurements below the World Health Organization Multicentre Growth Reference Study (WHO-MGRS) standard (weight for age in males, P=0.009, and females P=0.005). A delay in the initiation of breastfeeding was observed in female children (54.5%), showing a significant reduction in their weight (P=0.020) as compared to those initiated early. There was also a declining trend of continuation of breastfeeding from age 12 months to 23 months. The study revealed a high consumption of gripe water (68.8%) and bottle feeding (40.4%). A significant difference was found in children who consumed nutritious food, especially meat, with height (P=0.018) and weight (P=0.011), along with other foods.

Conclusion

IYCF and anthropometry have a direct association, evidence of which is reflected by the mother’s knowledge of feeding practices along with other socioeconomic parameters.

## Introduction

The health outcomes of a child are directly proportional to their feeding practices, which are, in turn, dependent on the knowledge and practices of the mother. The first two years of a child’s life are crucial to ensure appropriate growth and development. Malnutrition during this period results in a series of problems, beginning with reduced weight for age and stunting, progressing to the inability to achieve potential height in adulthood, and reduced capacity for physical work, which ultimately has implications for national development [1]. Improper feeding practices have also been linked to reduced reproductive capacity, complicated deliveries, and increased incidence of low birth weight infants in women who were malnourished as children [2].

The World Health Organization (WHO) has defined certain indicators to effectively assess infant and young child feeding practices. They are: early initiation of breastfeeding, exclusive breastfeeding under six months of age, continued breastfeeding at one year and at two years [3]. However, breastfeeding rates continue to be low worldwide, especially in high-income countries, where just one in five infants is breastfed [4]. A recent study analyzing the global trends on breastfeeding showed that the prevalence of exclusive breastfeeding among infants younger than six months in developing countries increased from 33% in 1995 to 39% in 2010 but has still not reached the target of at least 50% [5-6].

The National Family Health Survey (NFHS) of India is a multi-round survey conducted in a representative sample of households throughout the nation. The latest report of 2015-2016 (NFHS-4, India Fact Sheet) has determined that only 42.8% of urban neonates were given breast milk within one hour of birth and 52.1% urban infants were exclusively breastfed up to six months of age. It has also stated that the total number of urban children aged six to 23 months receiving an adequate diet is merely 11.6% [7].

Studies have been conducted to demonstrate the long-term effects of breastfeeding, with its impact on intelligence quotient (IQ) and prevention of diseases such as hypertension, type two diabetes mellitus, and even problems related to obesity [8-9]. It has also been shown to reduce the risk of pneumonia mortality and morbidity in young children [10].

This study was conducted to establish evidence of the association of infant and young child feeding (IYCF) with anthropometric measurements in an urban slum in Bangalore using the IYCF module by the United Nations Children’s Fund (UNICEF). Anthropometry measurements are simple and effective indicators of nutritional status and, hence, the adequacy of feeding practices.

Our primary objective was to study the feeding practices of infants and children from birth up to two years of age and the secondary objective was to study the association between feeding practices and anthropometric measurements among children of 12-23 months of age.

## Materials and methods

A community- based, cross-sectional study was conducted in April and May 2017 on mothers of 96 children who were between 12 and 23 months of age and who live in the urban slum setting of B.K. Nagar, Bangalore, Karnataka, India. This urban slum is part of the field practice area of MS Ramaiah Medical College, Bangalore, India. B.K. Nagar caters to a population of 96,000 and this area has been divided into census enumeration blocks (CEB) as per the government of India. Five such CEB were chosen at random and an orderly house-to-house survey was conducted. Mothers of children between 12 and 23 months of age were included as study subjects. Breastfeeding and complementary feeding information from these children from birth to 24 months was collected. Adopted children or children whose mothers were deceased were excluded.

Sample size

Based on the data from the latest report of NFHS-4 (2015-2016) results in India, which was obtained by multiple government field agencies that surveyed over 600,000 households, the expected proportion of urban neonates that were given breast milk within one hour of birth being 42.8% with an absolute precision of 10% and a confidence level of 95%, the required sample size was 94. This sample size was also adequate to study the second objective since children below five years of age and were stunted were 32.6% and those underweight for age were 31.5% according to NFHS-4 [7]. The ‘N MASTER 2.0 software’ (designed and developed by Biostatistics Resource and Training Centre, Christian Medical College, Vellore, India) was used to determine the above-mentioned sample size.

Permission to conduct the study was obtained from the institutional ethics board. On approval, informed consent was obtained from the study subjects explaining in detail about this research.

Study tool

The World Health Organisation has recently published a module titled "Indicators for assessing infant and young child feeding (IYCF) practices," which contains a questionnaire that was adapted to suit our study and helped us understand these practices [11]. The questionnaire begins with a household roster and other sociodemographic data to determine those subjects who are eligible for the questionnaire. The socioeconomic status for each family was calculated using the modified Kuppuswamy classification that was updated for 2017 with respect to income criteria [12]. The next section is the "initiation of breastfeeding module" which was administered to mothers of children aged 12-23 months. This was followed by the "infant and young child feeding module" which consists of questions regarding age, birth registration, and breastfeeding and complementary feeding details. After establishing a close rapport, a house to house survey was conducted per the recommendations of the IYCF module. The first and third author conducted the interview upon translating questions to the local language. Recall bias was minimized in the form of rephrasing questions to obtain consistency in responses.

Anthropometric measurements included weight, height, mid-upper arm circumference (MUAC), and head circumference (HC). Weight was documented using a weighing balance that was carried to the site (calibrated with a known weight every day) and measured to the nearest 0.5 kgs. Height was measured by making the child stand straight up on a leveled surface against the wall against a non-stretchable measuring tape. The MUAC was measured with a non-stretchable tape at a midpoint between the acromion and olecranon process of the non-dominant arm kept relaxed by the side of the body. HC was measured by placing the tape around the head over the glabella anteriorly and the occiput posteriorly by the overlap method. All parameters were measured to the nearest 0.5 cm.

Statistical analysis

Quantitative variables, such as age and anthropometric measurements, were analyzed using descriptive statistics such as mean and standard deviation. Qualitative variables, such as gender, infant breastfeeding, and supplementary feeding practices, were analyzed using frequencies, percentages, and the association of feeding practices with anthropometry was determined using percentages, Z scores, along with a 95% confidence interval. Tests used to determine significance were the student’s t-test, Z test (since the data followed a normal distribution), and chi-square test. Additionally, odds ratio (OR) was calculated to compare various food items with anthropometric data. The SPSS-18.0 software (SPSS Inc. Released 2009. PASW Statistics for Windows, Version 18.0. Chicago: SPSS Inc.) was used for basic statistical analysis.

In order to understand and compare the anthropometric data of this study population, the WHO Multicenter Growth Reference Study (MGRS) [13] was utilized as a standard of comparison. Additionally, WHO Anthro (Version 3.2.2, January 2011) was used to analyze these anthropometric parameters and generate graphs, which uses the MGRS data as a standard of comparison.

## Results

In this study, among 738 houses in five CEBs in the B.K. Nagar urban slum area, the first consecutive 94 households (96 children) that met the criteria were interviewed. It was found that there were 41 male children (42.7%) and 55 female children (57.3%), with more number of children in the age group of 21 to 23 completed months (28.1%). However, it was observed that the percentage of children in different age groups based on gender was not statistically significant (p=0.087). It was also found that the majority of the children were from a nuclear family and belonged to the upper lower socioeconomic group as shown in Table 1.

**Table 1:** Socio-demographic characteristics (total males = 41, total females = 55, N = 96) *: indicates values that are statistically significant

Socio-demographic characteristics	Males	Females	p-value
Age in months			
12-14	12 (29.3%)	9 (16.4%)	
15-17	8 (19.5%)	8 (14.5%)	
18-20	8 (19.5%)	24 (43.6%)	
21-23	13 (31.7%)	14 (25.5%)	
Total	41 (100%)	55 (100%)	0.087
Type of family			
Nuclear	28 (68.3%)	34 (61.8%)	
Joint	6 (14.6%)	9 (16.4%)	
Three generation	7 (17.1%)	12 (21.8%)	
Total	41 (100%)	55 (100%)	0.793
Socioeconomic Status {12}			
Upper	4 (9.8% )	2 (3.6%)	
Upper middle	12 (29.3%)	14 (25.5%)	
Lower middle	8 (19.5%)	15 (27.3%)	
Upper lower	17 (41.5%)	21 (38.2%)	
Lower	0 (0%)	3(5.5%)	
Total	41 (100%)	55 (100%)	0.519
Maternal Education			
Up to Middle School	9 (69.2%)	4 (30.8%)	
High School	19 (32.8%)	39 (67.2%)	
Above High School	13 (52.0%)	12 (48.0%)	
Total	41 (100%)	55 (100%)	0.031*
Maternal occupation			
Housewife	33 (80.5%)	50 (90.9%)	
Employed	8 (61.5%)	5 (38.5%)	
Total	41 (100%)	55 (100%)	0.140

**Figure 1:**
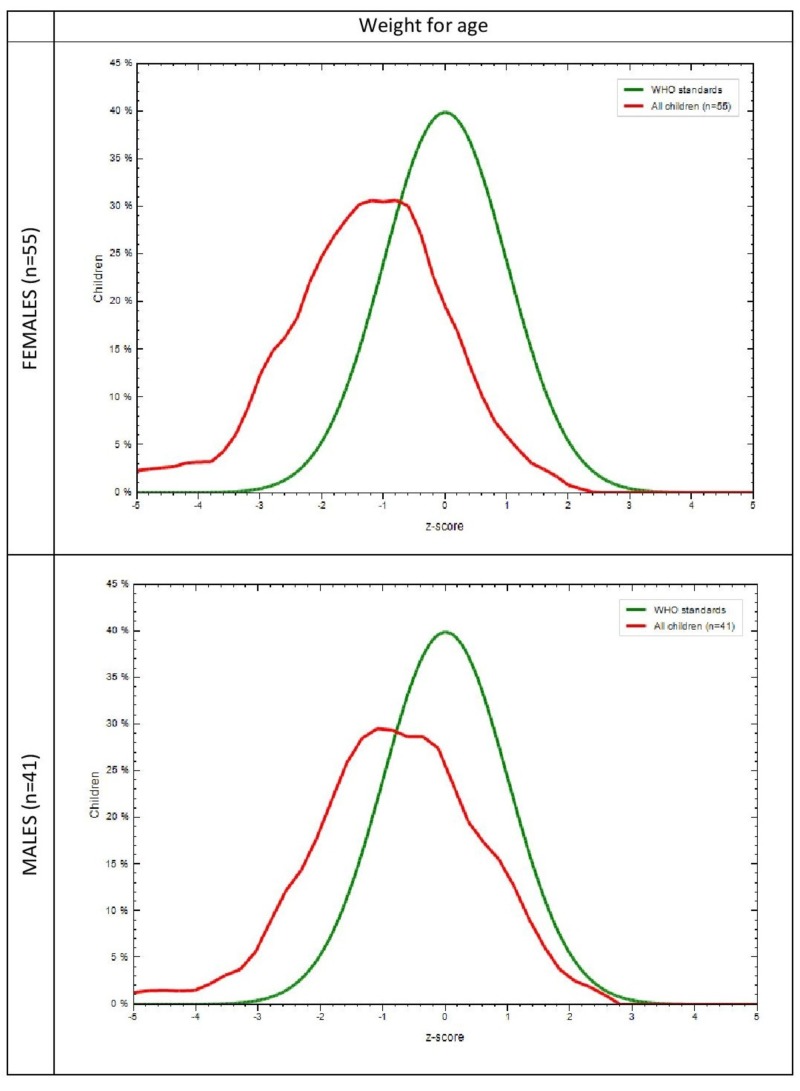
Comparison of weight for age in the study group and the WHO-MGRS standard by gender WHO-MGRS: World Health Organization Multicenter Growth Reference Study X-axis represents z scores Y-axis represents % of children Green curve represents the WHO standard Red curve represents the study population

**Figure 2:**
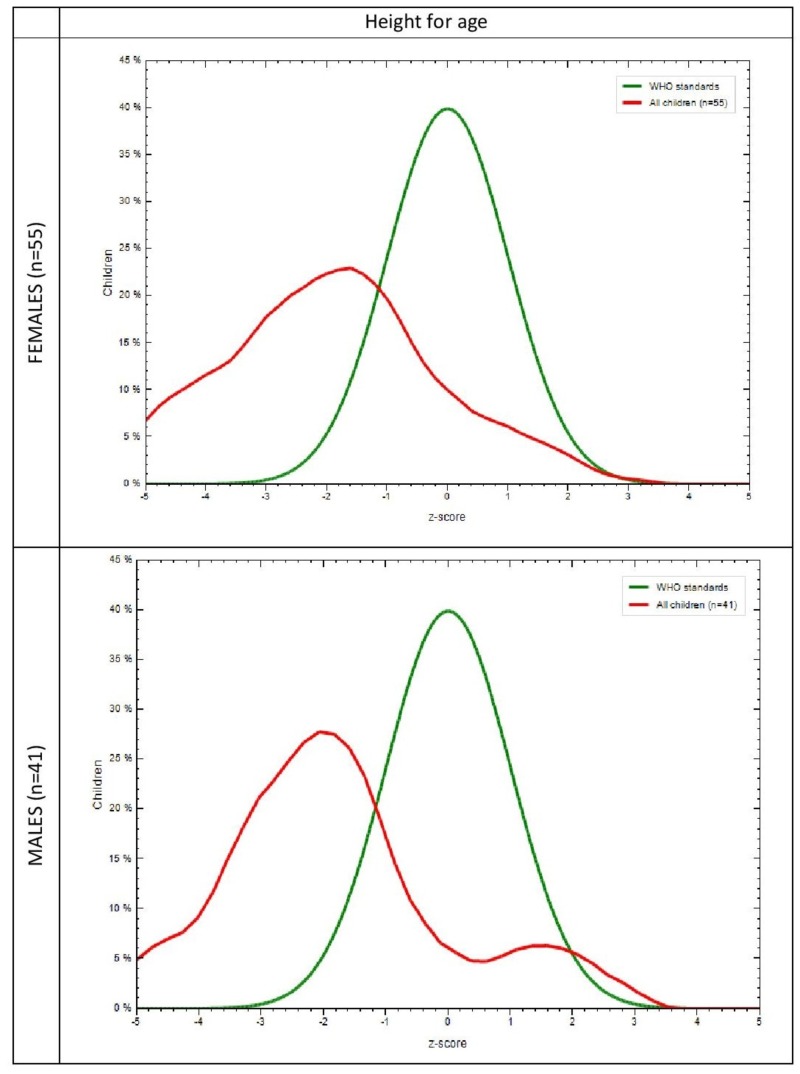
Comparison of height for age in the study group and the WHO-MGRS standard by gender WHO-MGRS: World Health Organization Multicenter Growth Reference Study X-axis represents z scores Y-axis represents % of children Green curve represents the WHO standard Red curve represents the study population

**Figure 3:**
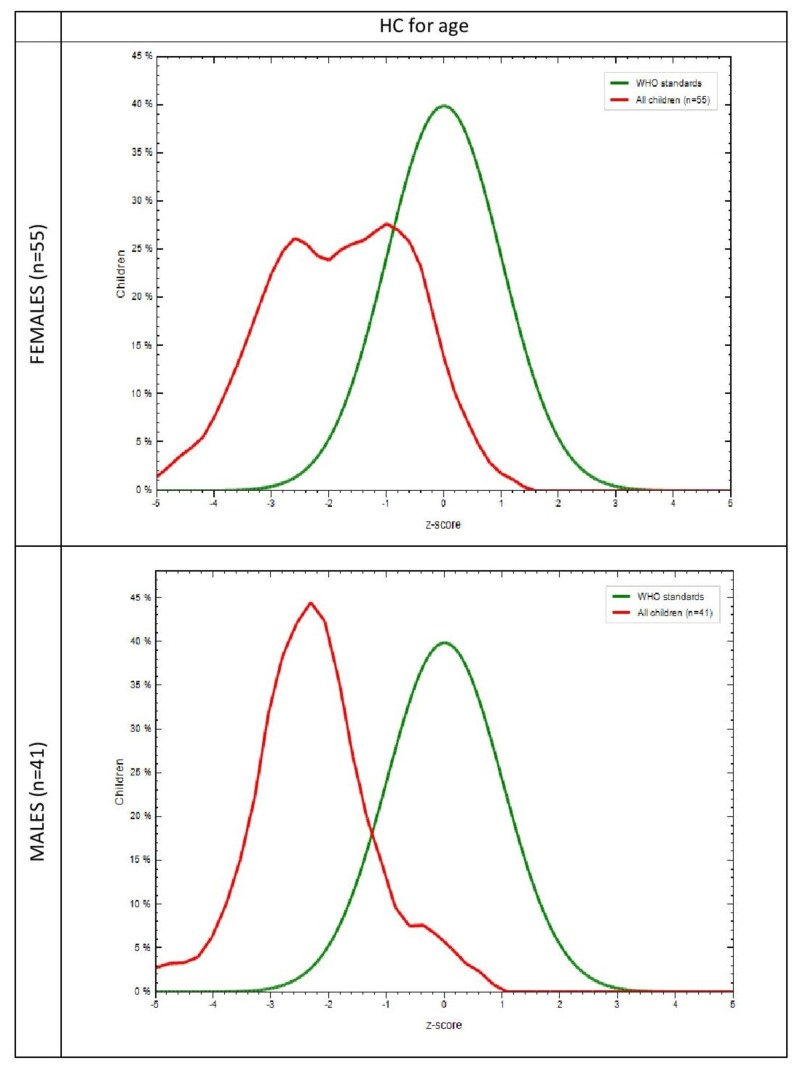
Comparison of head circumference for age in the study group and the WHO-MGRS standard by gender WHO-MGRS: World Health Organization Multicenter Growth Reference Study; HC: Head circumference X-axis represents z scores Y-axis represents % of children Green curve represents the WHO standard Red curve represents the study population

**Figure 4:**
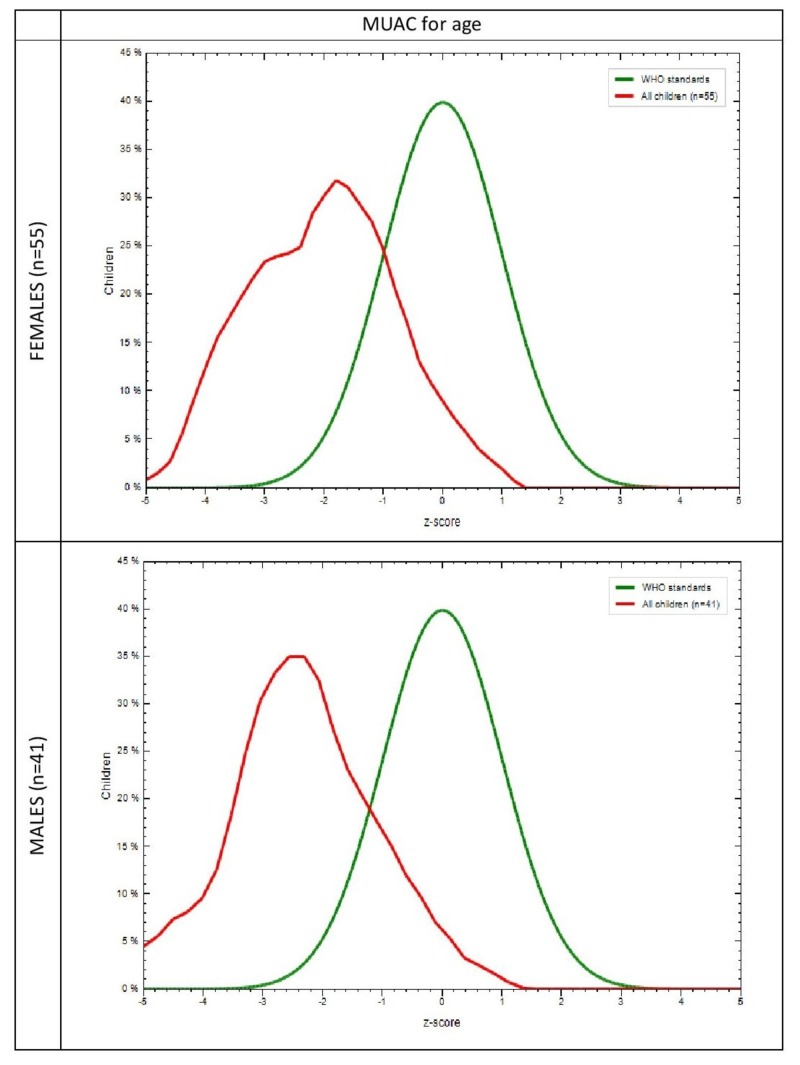
Comparison of mid-upper arm circumference for age in the study group and the WHO-MGRS standard by gender MUAC: Mid-upper arm circumference; WHO-MGRS: World Health Organization Multicenter Growth Reference Study X-axis represents z scores Y-axis represents % of children Green curve represents the WHO standard Red curve represents the study population

The mean anthropometric measurements of the study population are shown in Table 2. A difference in weight, height, and HC between males and females, notably in the age group of 21-23 months (p=0.006, p=0.047, p=0.001) were found to be significant. Anthropometric measurements in the study group and the WHO-MGRS standard with respect to gender was plotted graphically. It was observed that in the study population, the mean Z scores of 30% females (p=0.005) and 28% males (p=0.009) for weight for age (Figure 1), 24% females (p=0.006), and 27% males (p=0.061) for height for age (Figure 2) were lower than the mean Z scores of the WHO population, which was 40%. It was also found that 26% females (p=0.018) and 45% males (p=0.589) for HC for age (Figure 3) and 32% females (p=0.204) and 36% males (p=0.596) for MUAC for age (Figure 4) also followed the same trend. WHO Anthro was used to analyze the basic indicators of malnutrition and the cut-off for each indicator was considered to be below -2 SD. It was observed that out of the total population, 16.7% (males = 9.8%, females = 21.8%) were wasted, out of which 4.2% (males 2.4%, females = 5.5%) were severely wasted (below -3 SD), 50 % (males = 56.1%, females = 45.5%) stunted and 26% (males = 14.6%, females = 34.5%) underweight.

**Table 2:** Mean (± SD) anthropometric measurements *: indicates values that are statistically significant MUAC: mid-upper arm circumference HC: head circumference

Anthropometric measure	Age group in months	Male (Mean ± SD)	Female (Mean ± SD)	p-value
Weight in kgs				
	12-14	8.67 ± 1.231	7.67 ± 1.000	0.061
	15-17	10.63 ± 1.664	9.25 ± 1.909	0.147
	18-20	10.00 ± 1.512	9.50 ± 1.251	0.360
	21-23	11.08 ± 1.320	9.29 ± 1.729	0.006*
				0.001*
Height in cms				
	12-14	71.58 ± 4.379	71.33 ± 5.701	0.911
	15-17	77.88 ± 3.871	76.13 ± 6.999	0.546
	18-20	79.25 ± 6.182	76.01 ± 5.393	0.166
	21-23	79.08 ± 3.278	76.00 ± 4.261	0.047*
				0.041*
MUAC in cms				
	12-14	12.083 ± 1.311	12.200 ± 1.3039	0.842
	15-17	13.00 ± 0.845	11.750 ± 0.845	0.010*
	18-20	12.625 ± 0.91	12.583 ± 1.070	0.922
	21-23	12.50 ± 1.2910	12.607 ± 1.288	0.831
				0.292
HC in cm				
	12-14	43.29 ± 1.373	43.22 ± 1.481	0.913
	15-17	44.25 ± 0.655	43.88 ± 1.885	0.603
	18-20	44.00 ± 1.309	44.35 ± 1.485	0.553
	21-23	45.69 ± 1.316	43.54 ± 1.538	0.001*
				0.354

Initiation of breastfeeding is recommended as soon as the mothers give birth [1]. Early initiation of breastfeeding is important; however, certain circumstances may limit the accessibility of a child to breast milk due to various reasons (Table 3). Irrespective of the method of delivery, initiation of breastfeeding up to one hour was considered as not delayed. On analysis of the children’s feeding practices, it was found that the delay in breastfeeding was more in female children (54.5%) as compared to male children (43.9%) with the most common reason being a delay in initiation after a cesarean section (Table 3). It was also observed that 3.6% of the female children were not initiated with breast milk early due to religious reasons. A comparison of mean anthropometric measurements amongst the "delayed" and "not delayed" revealed that there was a significant reduction in weight among females in whom breastfeeding was delayed (p=0.020) (Table 4). In terms of continuity of breastfeeding, it was found that at age 12 months 84.6%, at 18 months 50%, and at 23 months only 14.3% of children were given breast milk along with complementary foods.

**Table 3:** Breastfeeding practices and reasons for delay *: indicates values that are statistically significant PPH: Post-partum hemorrhage NICU: Neonatal intensive care unit

Initiation of Breastfeeding		Males	Females	P value
Delayed	1 – 3 hours	9 (50%)	21 (70%)	
	> 3 hours	9 (50%)	9 (30%)	
Total		18(43.9%)	30 (54.5%)	0.106
Not delayed		23 (56.1%)	25 (45.5%)	
Total		41 (100%)	55 (100%)	0.302
Reasons for delay				
C section		9 (22.0%)	14 (25.5%)	
No milk production		4 (9.8%)	5 (9.1%)	
PPH postoperative complications		1 (2.4%)	3 (5.5%)	
NICU admission of child		2 (4.9%)	1(1.8%)	
Religious reasons		0 (0%)	2 (3.6%)	
Feeding issues with child		2 (4.9%)	0 (0%)	
No specific reason/Don’t know		0 (0%)	5 (9.1%)	
Total		18 (100%)	30 (100%)	0.201

**Table 4:** Mean anthropometry by initiation of breastfeeding *: indicates values that are statistically significant MUAC: Mid-upper arm circumference HC: Head circumference

Anthropometry	Males			Females		
	Delayed	Not delayed	P value	Delayed	Not delayed	P value
Weight in kgs	9.72 ± 1.555	10.35 ± 1.748	0.240	8.67 ± 1.729	9.64 ± 1.150	0.020*
Height in cm	77.22 ± 5.440	76.26 ± 5.437	0.578	74.71 ± 5.445	75.92 ±5.751	0.426
MUAC in cm	12.556 ± 1.069	12.457 ± 1.251	0.099	12.450 ± 1.241	12.352 ± 1.0576	0.757
HC in cm	44.58 ± 1.478	44.22 ± 1.594	0.366	43.63 ± 1.727	44.20 ± 1.354	0.188

The IYCF questionnaire includes details of complementary feeds given to the child, though it does not include the quantity of food consumed and age of initiation. Food items were analyzed in terms of liquids and semisolids consumed and the results were as follows. Among liquids, 15.6% of the study group consumed formula feeds and 94.8% of the population consumed buffalo’s/cow’s milk with 69.8% consumption of other dairy products. Other liquids consumed included fruit juices (70.8%), gripe water (68.8%), oral rehydration salts (17.7%), and vitamin drops (13.5%). Among semisolid food items, cereals, roots, tubers, fruits, and vegetables were consumed by all children. It was found that 86.5% of the study population were non-vegetarians (including egg) and 13.5% were pure vegetarians. Among the non-vegetarians, the percentage of consumption was as follows: organ meat 24.0%, meats 64.6%, eggs 81.3%, and seafood 53.1%. Foods rich in vitamin A were consumed by 93.8% of the population. The percentage consumption of oils was 74% and that of sugary food and condiments were 93.8% and 64.6%, respectively. On detailed questioning of feeding practices, it was observed that 40.6% of the study population provided bottle feeds to their children, with the rate of bottle feeding increasing from 23.8% at 12-14 months to 51.9% at 21-23 months.

Comparison of mean anthropometric measurements of those consuming a particular food item and those who did not were made. Though all measurements and food items were analyzed, a significant difference was found only with weight and height among those who consumed meat, eggs, seafood, sugary food, gripe water, thin porridge, and clear broth, as shown in Tables 5-6. The differences in HC and MUAC among study population who consumed the above-mentioned food items were not found to be statistically significant. There was also no statistical difference among those who consumed cereals, grains, foods rich in vitamin A, fruits, green leafy vegetables, roots, tubers, and milk products.

**Table 5:** Mean (±SD) weight (in kg) of children aged 12-23 months based on consumption of each food item *: indicates values that are statistically significant

		Total population (n=96)		Males (n=41)		Females (n=55)	
Complementary food	Consumption	Mean ± SD	p-value	Mean ± SD	p-value	Mean ± SD	p-value
Meat	Consumed	9.84 ± 1.642	0.011*	10.67 ± 1.711	0.005*	9.32 ± 1.378	0.143
	Not consumed	8.94 ± 1.590		9.24 ± 1.239		8.65 ± 1.869	
Eggs	Consumed	9.73 ± 1.678	0.010*	10.27 ± 1.750	0.123	9.33 ± 1.523	0.022*
	Not consumed	8.61 ± 1.335		9.25 ± 1.035		8.10 ± 1.370	
Seafood	Consumed	9.84 ± 1.602	0.044*	10.20 ± 1.601	0.642	9.61 ± 1.585	0.005*
	Not consumed	9.16 ± 1.692		9.95 ± 1.774		8.46 ± 1.285	
Sugary food	Consumed	9.48 ± 1.656	0.331	10.13 ± 1.737	0.360	8.98 ± 1.503	0.027*
	Not consumed	10.17 ± 1.941		9.00 ± 2.828		10.75 ± 1.500	
Gripe water	Consumed	9.74 ± 1.542	0.054*	10.13 ± 1.737	0.714	9.38 ± 1.256	0.099
	Not consumed	9.03 ± 1.861		9.89 ± 1.516		8.67 ± 1.906	
Thin porridge	Consumed	8.81 ± 1.358	0.000*	9.35 ± 1.101	0.019*	8.50 ± 1.408	0.001*
	Not consumed	10.20 ± 1.671		10.58 ± 1.840		9.84 ± 1.438	
Clear broth	Consumed	9.49 ± 1.683	0.330	10.00 ± 1.711	0.506	9.13 ± 1.579	0.845
	Not consumed	9.69 ± 1.653		10.50 ± 1.517		9.00 ± 1.528	

**Table 6:** Mean (± SD) height (in cm) of children aged 12-23 months based on the consumption of each food item *: indicates values that are statistically significant

		Total population (n=96)		Males (n=41)		Females (n=55)	
Complimentary food	Consumption	Mean ± SD	P value	Mean ± SD	p-value	Mean ± SD	P value
Meat	Consumed	74.84 ± 5.068	0.018*	77.75 ± 5.447	0.134	76.27 ± 4.799	0.043*
	Not consumed	74.09 ± 5.905		75.18 ± 5.090		73.00 ± 6.595	
Eggs	Consumed	76.54 ± 5.289	0.012*	77.42 ± 5.196	0.073	75.89 ± 5.320	0.072
	Not consumed	72.94 ± 5.641		73.63 ± 5.423		72.40 ± 6.041	
Seafood	Consumed	76.80 ± 5.040	0.076	76.55 ± 5.365	0.880	76.97 ± 4.902	0.008*
	Not consumed	74.80 ± 5.875		76.81 ± 5.546		73.05 ± 5.691	
Sugary food	Consumed	75.69 ± 5.420	0.229	76.82 ± 5.375	0.478	74.83 ± 5.345	0.039*
	Not consumed	78.50 ± 6.716		74.00 ± 7.071		80.75 ± 6.185	
Gripe water	Consumed	76.64 ± 4.974	0.041*	76.88 ± 5.885	0.673	76.42 ± 4.011	0.048*
	Not consumed	74.17 ± 6.298		76.00 ± 3.240		73.38 ± 7.152	
Thin porridge	Consumed	73.56 ± 5.261	0.000*	74.94 ± 4.683	0.082	72.77 ± 5.482	0.000*
	Not consumed	78.08 ± 4.830		77.92 ± 5.610		78.24 ± 4.055	
Clear broth	Consumed	75.57 ± 5.105	0.182	75.60 ± 4.717	0.001*	75.55 ± 5.420	0.320
	Not consumed	77.77 ± 7.596		83.00 ± 5.020		73.29 ± 6.601	

Odds of above-average weight and height by the consumption of each food item was calculated by gender. The mean weight of male children who consumed meat was significantly higher when compared to those who did not (OR=6.533; p=0.009). The mean weight of female children who consumed seafood was significantly higher when compared to those who did not (OR=4.154; P=0.014). In terms of height, the mean height of male children who consumed peas (OR=7.438, P=0.046) was significantly higher whereas the mean height of female children who consumed meat and were fed with gripe water (OR=3.680, p=0.033, OR=3.23, p=0.02) was significantly higher as compared to those who did not. The other food items did not show any statistical significance with respect to increased weight or height.

## Discussion

This study was mainly focused on establishing an association between infant and young child feeding practices and anthropometric measurements in an urban slum population in Bangalore, India. Although similar studies to demonstrate the association between feeding practices and nutritional status were done in rural areas, this UNICEF questionnaire was not used [14-16]. Further, our study provides detailed descriptive data regarding types of complementary food given to children in an urban slum population.

In this study, the male to female ratio is 0.74:1, with a female child preponderance. Hence, all analyses have been made according to gender and age. Our study did not reveal any statistical significance among the percentage of children in different age groups with respect to gender (Table 1).

In an economically and culturally diverse country such as India, differences in socioeconomic status, family structure, maternal education, and occupation can shed some light on infant and young child feeding practices [17]. Our study revealed that most families belonged to a nuclear family structure and to the upper lower socioeconomic category. In terms of maternal education, the majority of the mothers who were interviewed were housewives (86.4%) and most mothers had studied up to a high school level, showing a significant statistical difference between the mothers of male and female children (Table 1). In a similar study with a female preponderance, half of their study population belonged to nuclear families and 91.8% of the mothers were housewives [15]. In another study conducted in Karnataka, it was observed that 51% of the population were from joint families and 49% nuclear families, but a majority of their study population belonged to the upper lower socioeconomic status group, similar to our study [18]. However, the primary focus of both these papers was on a rural population and studies focusing on urban slum populations in India are quite limited.

It is vital to understand the family structure, mother’s occupation, and family socioeconomic status, as it directly has an effect on the nutritional status of infants and young children. In an urban slum population, nuclear families are predominant over extended families probably due to a limitation in space and housing facilities and the increased cost of living. This limits alternative childcare support, such as grandparents or cousins, especially needed for working parents, which is the case with nuclear families in lower socioeconomic groups. It has been observed that working mothers have reported difficulty in feeding their children notably by skipping breastfeeding or storing expressed milk, which in turn results in untimely feeds to the child. Furthermore, this study also notes that mothers find difficulty in cooking; thus increasing the likelihood of providing readymade food of poor nutritional value to their children. There also exists a lack of diversity of food items thus reducing net food intake by the child. Comparatively, mothers in extended families report ease in feeding their children, as they rely on siblings and other relatives. However, there exists a risk of untimely feeding practices and possible lack of knowledge of nutritious food choices [19].

In our study population, early initiation of breastfeeding was found to be more in males (56.1%) as compared to females (45.5%) (Table 3). The NFHS-4 fact sheet reports the early initiation of breastfeeding in the urban population as 42.8% and 53.7% for India and Karnataka [7,20]. Various studies have indicated a gender bias with respect to infant and young child feeding with male children being given more importance than females, more so in rural areas [14,18,21-22]. A total of 50 % of the mothers reported a delay in the initiation of breastfeeding. However, only a small proportion delayed breastfeeding beyond three hours though results were not statistically significant. The most common reason for the delay was found to be due to cesarean section deliveries, followed by inadequate milk production (Table 3). In a similar study in South India, it was found that in 40.4% of the children, initiation of breastfeeding was within one hour of birth and 29.6% of mothers initiated feeding within the first four hours of birth. This study also lists reasons for the delay in the initiation of breastfeeding in descending order as maternal surgery (27.6%), lack of milk production (21.5%) and religious reasons (18.2%). However, the study also did not show any statistical significance between the initiation of breast milk and gender [23].

In our study group, 84.6%, 50%, and 14.3% of mothers at 12, 18, and 23 months respectively, continued breastfeeding. Possible reasons for discontinuing breastfeeding could be the cessation of milk production, lack of knowledge regarding the continuation of breastfeeding, and the ability of the child to quickly adjust to complementary food. However, two other studies showed the continuation of breastfeeding at one and two years of age as 99.7% and 87.2% and 88.1% and 73.1%, respectively [16,24]. In order to improve the nutritional status of our children, it is essential to create awareness among mothers regarding the advantages of continued breastfeeding particularly in lower socioeconomic groups [25].

The proportion of urban slum children who were fed with a bottle was 40.6% whereas it was 51.9% in the 21-23 months age group. Studies in various states of India such as Karnataka, Jammu and Kashmir, and Punjab show similar proportions of bottle feeding: 49.4%, 51.5%, and 63.3%, respectively [16,26-27].

The MGRS [13] was designed to provide a single international standard by the collection of anthropometric data from children representative of various ethnic backgrounds and socioeconomic status and, hence, was used to assess our subjects as well. The graphs that were generated in comparison with the MGRS population showed that the mean Z scores for all anthropometric measurements in our subjects were less than that of the standard (Figure 1). Since racial, ethnic, and socioeconomic differences have been addressed in the MGRS, nutrition is probably responsible for such low means in our study population. This further reinforces the fact that early initiation of breastfeeding, continued breastfeeding, and adequate complementary feeding can, in fact, result in better nutritional outcomes evidenced by better mean anthropometric parameters.

In our study, we found that 16.7% were wasted, out of which 4.2% were severely wasted (below -3 SD), 50% stunted, and 26% underweight. The NFHS - 4 for India reports 20.0% wasted out of which 7.5% were severely wasted, 31.0% stunted, and 29.1% underweight in children below five years of age [7]. Similarly, statistics from the Karnataka fact sheet shows 24.8% wasted out of which 9.7% were severely wasted, 32.6% stunted, and 31.5% underweight in children below five years of age [20].

An attempt at formulating an association between the type of complementary food consumed and each anthropometric measurement was made and a positive impact on the weight and height parameters were observed, particularly with some food items, such as meat, eggs, seafood, thin porridge, with certain results showing statistical significance (Tables 5-6). Most studies have analyzed the consumption of various complementary food items independent of the anthropometric measurements of their study population and thus have not been able to show a direct association between the two [24,26,28-29]. In our study, we have compared the proportion of food consumption with height and weight as a 1:1 comparison, unlike previous studies. It can, however, be argued that the exact quantity of food consumed was not taken into consideration, the lack of which limits in-depth understanding of IYCF, which can be implemented in future studies for a precise analysis.

Gripe water consumption and its effect on the nutritional status of a child has always been a controversial topic of discussion and was addressed in this study. It was found that the height and weight of the total population were significantly higher in those children who consumed gripe water as compared to those who did not (p=0.041, p=0.054). Gripe water is most commonly used as a pre-lacteal feed in most children, but in our study, we found that mothers went on to give their children gripe water along with complementary feeding. The most common reasons for using gripe water was to aid in digestion, insistence by elders, and to relieve abdominal colic though studies have shown that gripe water, in fact, has resulted in an increase in constipation, infantile colic, fevers, cough and cold, and diarrhea [30].

The urban slum undertaken in this study is part of the field practice area of our teaching hospital, which made it easier for the investigators to establish a good rapport with its residents. Most studies with similar objectives were conducted in a rural population and indigenously designed questionnaires were used. In our study, we utilized the UNICEF IYCF questionnaire in an urban slum population. Meticulous collection of data was achieved by using random sampling of CEBs for minimizing the selection bias and rephrasing questions to reduce the recall and information bias. This study is distinct from other studies in terms of comparing the consumption of complementary food with anthropometric measurements to show a possible direct cause-effect relationship, unlike other studies that have studied the same parameters independently.

Even though the latest NFHS fact sheets were taken into consideration while calculating the required sample size, our sample size was small. Larger sample size would help in demonstrating stronger associations between IYCF and anthropometry. In terms of study design, a follow-up study would have been more robust with respect to reducing the recall bias, measurement bias by inquiring and verifying the quantity of food given, interval anthropometric measurements, and information regarding illnesses if occurred during the study period that could alter feeding and anthropometry. The survey lacks questions on the duration of exclusive breastfeeding and age at initiation of complementary feeds required for an in-depth understanding of IYCF. The effect of gripe water on the anthropometric measurements of infants and young children needs to be further studied.

## Conclusions

Infant and young child feeding practices are one of the most commonly studied aspects of maternal knowledge of child nutrition, yet many countries still observe a significant amount of malnutrition and ill health among its children. Knowledge regarding feeding practices, commencing from the initiation of breastfeeding to complementary feeding, especially during the early growing years, is vital to overall child development. In this study, we have also addressed the importance of the continuation of breastfeeding for up to two years of age and this must be advocated to families at primary health care centers and health camps. An attempt to understand the correlation between basic food items and anthropometry was also done in this study, which revealed the necessity of further such correlation studies. This, in our opinion, would help social workers and health care professionals alike to guide families in raising strong and healthy children. In the long term, this will prove beneficial to not only the community but eventually overall national development as well.
